# Clinicians' psychological empowerment to engage in management as part of their daily work

**DOI:** 10.1108/JHOM-08-2021-0300

**Published:** 2022-10-13

**Authors:** Thomas Andersson, Nomie Eriksson, Tomas Müllern

**Affiliations:** School of Business , University of Skövde , Skövde, Sweden; Jönköping International Business School , Jönköping, Sweden; Faculty of Theology, Diaconia and Leadership Studies , VID Specialized University , Oslo, Norway

**Keywords:** Psychological empowerment, Physician, Nurse, Managerial work, Integration

## Abstract

**Purpose:**

The purpose of the article is to analyze how physicians and nurses, as the two major health care professions, experience psychological empowerment for managerial work.

**Design/methodology/approach:**

The study was designed as a qualitative interview study at four primary care centers (PCCs) in Sweden. In total, 47 interviews were conducted, mainly with physicians and nurses. The first inductive analysis led us to the concept of psychological empowerment, which was used in the next deductive step of the analysis.

**Findings:**

The study showed that both professions experienced self-determination for managerial work, but that nurses were more dependent on structural empowerment. Nurses experienced that they had competence for managerial work, whereas physicians were more ignorant of such competence. Nurses used managerial work to create impact on the conditions for their clinical work, whereas physicians experienced impact independently. Both nurses and physicians experienced managerial work as meaningful, but less meaningful than nurses and physicians' clinical work.

**Practical implications:**

For an effective health care system, structural changes in terms of positions, roles, and responsibilities can be an important route for especially nurses' psychological empowerment.

**Originality/value:**

The qualitative method provided a complementary understanding of psychological empowerment on how psychological empowerment interacted with other factors. One such aspect was nurses' higher dependence on structural empowerment, but the most important aspect was that both physicians and nurses experienced that managerial work was less meaningful than clinical work. This implies that psychological empowerment for managerial work may only make a difference if psychological empowerment does not compete with physicians' and nurses' clinical work.

## Introduction

This paper addresses whether clinical physicians' and clinical nurses' psychological empowerment to engage in management as part of their daily work could make managerial work and clinical work in health care organizations better integrated. Health care organizations have always been considered difficult to manage despite numerous reforms to make them more manageable. The main explanation of the difficulties is the high complexity of health care, which can be explained by multiple co-existing, often competing institutional logics ( Martin *et al.* , 2017 ). The strongest conflict tends to be between managerial logic of health care managers and professional logic of physicians ( Andersson and Liff, 2018 ), but there are many more logics in play that further increase complexity, such as professional logic of nurses and other professions and community logics of politicians ( van den Broek *et al.* , 2014 ; Currie and Spyridonidis, 2016 ). However, the main problem is not that there are different logics in health care organizations, but that they are poorly integrated with each other ( Andersson, 2022 ). Institutional logics guide individual behavior, but since clinicians and managers in health care tend to adhere mainly to different institutional logics ( Andersson and Liff, 2018 ; Martin *et al.* , 2017 ), the differences in the logics create conflict in the workplace between them because of different values, priorities, and focus.

Clinicians and managers both represent important values for high-quality, effective health care ( Degeling *et al.* , 2003 ), so the inability to solve such problems reduces the quality and efficiency of health care. The most common way to approach the problem is to make structural changes, with the aim of integrating physicians into administrative structures ( Baker and Denis, 2011 ; Liff and Andersson, 2011 ), which basically means trying to embody the two logics in one person ( Dawson *et al.* , 1995 ) and let clinicians lead in hospitals ( Snell *et al.* , 2011 ; Witman *et al.* , 2011 ). However, there is an imbalance in these hybrid forms, and most clinicians primarily remain clinicians ( Llewellyn, 2001 ; Andersson, 2015 ). Also such structural changes offer no guarantee of a balance among different logics ( McGivern *et al.* , 2015 ) because of clinicians' limited engagement in managerial logics ( Llewellyn, 2001 ). However, nurses appear to have been easier to involve in managerial work than physicians ( Ernst, 2019 ).

Another less-researched approach to the problem is to make clinicians engage in managerial work without structural changes, i.e. without clinicians entering managerial positions ( Andersson and Liff, 2018 ). An example is professional hybrids ( Blomgren and Waks, 2015 ) who are experienced clinicians who engage in managerial work, but remain in clinical positions. Such hybrids have proven to be able to translate between the worlds of medicine and management and can thereby bridge other clinicians and managers ( Blomgren and Waks, 2015 ). These are rare, however, which illustrates Bååthe and Norbäck's (2013) claim that clinicians in general have not developed any greater appreciation of managerial processes. There is little practical proof that clinicians actually are involved in or appreciate managerial logic ( Adams *et al.* , 2020 ). Instead, clinicians seem reluctant to engage in managerial work as part of their daily practices. To understand this better, we direct our attention to Noordegraaf (2015) , who claimed that professionals should be *empowered* to deal with work that not only involves professional logic, but also managerial logic. However, there is a lack of empirical research on whether clinicians in health care are capable, ready, and willing to connect, engage, and involve more in managerial logic; this empirical quest is the focus of this paper.

In practice, managerial logic is not normally encountered in an ideal or abstract state, but manifests within the particularities of everyday work ( Bechky, 2011 ). Manifestations of managerial logic include managerial work activities, which are not only the work of managers, but also of clinicians who do not hold managerial roles (cf. Tengblad, 2012 ). Thus, managerial work includes more administrative and coordinative activities related to a resource perspective and a development perspective that is not clinically based ( Wikström and Dellve, 2009 ). Clinicians' *empowerment* for managerial work may lead to possibilities to balance different contradictory roles ( van Schothorst-van Roekel *et al.* , 2020 ) and thus integration of health care organizations, but it needs empirical investigations, since we lack knowledge regarding if and how clinicians experience empowerment for managerial work.

The concept of empowerment can be divided into *structural empowerment* ( Kanter, 1993 ), which focuses on how the organizational structure provides power, and *psychological empowerment* , which focuses on individuals' perceptions of being in control of their work (e.g. Conger and Kanungo, 1988 ; Spreitzer, 1995 ; Thomas and Velthouse, 1990 ). The division is important because it illustrates that structural empowerment is a necessary, but not sufficient, antecedent to psychological empowerment ( Maynard *et al.* , 2012 ). Structural empowerment needs to precede psychological empowerment, but is no guarantee for it. In the present study, while structural empowerment is a prerequisite for selected empirical cases, our focus is on psychological empowerment. The aim is to analyze how physicians and nurses, as the two major health care professions, experience psychological empowerment for managerial work.

We use a model that identifies four general cognitions of psychological empowerment ( Spreitzer, 1995 ; Thomas and Velthouse, 1990 ) and adapt it to describe psychological empowerment among physicians and nurses in relation to managerial work. The potential of this aim is for psychological empowerment to serve as a way to integrate health care clinicians and managers and thus professional logics and managerial logic, if it means that clinical staff engage to a higher degree in what could be labeled managerial work.

## Previous research on empowerment of clinical staff in health care for managerial work

Most previous research on how physicians and nurses may support integration in health care has focused on physicians and nurses who hold managerial positions, but with rather disappointing results regarding integration (e.g. Andersson, 2015 ; Llewellyn, 2001 ; McGivern *et al.* , 2015 ). Our focus is on how clinical physicians and nurses (non-managers) experience empowerment for managerial work. Consequently, we focus on managerial work among clinicians rather than managers' work (e.g. Tengblad, 2012 ), but previous research is limited. Empowerment research involving physicians and nurses almost exclusively concern empowerment for their clinical work (e.g. Leggat *et al.* , 2010 ; Wagner *et al.* , 2010 ).

In previous research, empowerment has been divided into structural and psychological empowerment ( Kanter, 1993 ), where structural empowerment means that power (and therefore responsibilities and tasks) that originates in managerial positions is already delegated to non-managerial positions. Research has shown that structural empowerment is a prerequisite for good health care practices ( Lankshear *et al.* , 2013 ) and the most common empowering structure in health care organizations is teams ( Proenca, 2007 ). Psychological empowerment is the individuals' experiences of the structural empowerment and focuses on the perceptions of being in control of their work ( Conger and Kanungo, 1988 ).

Several studies have confirmed that psychological empowerment leads to increased quality of patient care quality and organizational effectiveness in health care (e.g. Bonias *et al.* , 2010 ; Knol and van Linge, 2009 ; Laschinger *et al.* , 2002 ; Leggat *et al.* , 2011 ; Malak and Abu Safieh, 2022 ); however, since most research is quantitative and correlational, we have limited information about *how* psychological empowerment influences quality of patient care and organizational effectiveness. As Lloyd *et al.* (1999) emphasized more than 20 years ago, empowerment is a complex concept, especially in a health care context. Therefore, there is a need for more qualitative research to understand the mechanisms that explain how psychological empowerment and organizational effectiveness are related.

Empirical results indicate the importance of clinicians' psychological empowerment for managerial work. In a major investigation of psychological empowerment in health care, Bonias *et al.* (2010) concluded that quality of patient care is not only about the pure clinical activities, but also depends on other integrated activities. Those authors did not investigate managerial activities *per se* , but they did confirm the importance of activities other than purely clinical ones for quality of patient care (such as managerial work). Similarly, Leggat *et al.* (2010) , Leggat *et al.* (2011) and McAlearney *et al.* (2011) indicated the importance of psychological empowerment for managerial work. They emphasized that structural empowerment through organizational initiatives such as decentralization must be followed by unit managers acting accordingly. The consequence of these results should be that unit managers who decentralize their activities need employees to take on these managerial activities and experience psychological empowerment for managerial work. Their study confirms the employees' psychological empowerment in general, but not explicitly in relation to managerial work. Similarly, Bobbio *et al.* (2012) and Al Otaibi *et al.* (2022) confirmed the importance of empowering leadership in health care, which indicates that employees are empowered to lead their own work. All six studies ( Al Otaibi *et al.* , 2022 ; Bobbio *et al.* , 2012 ; Bonias *et al.* , 2010 ; Leggat *et al.* , 2010 , 2011 ; McAlearney *et al.* , 2011 ) emphasized the importance of investigating psychological empowerment as an effect of organizational initiatives to support empowerment; in other words, micro-level studies of employees are important for understanding the effects of organizational initiatives such as high-performance work systems (HPWS). Recent research (e.g. as described above, research on psychological empowerment in health care commonly) investigates psychological empowerment in general, and not particularly in relation to managerial work, but it is possible to find indications from these general studies that psychological empowerment for managerial work is important. One study that has a similar focus as ours is Müllern and Nordin (2012) , but they used psychological empowerment to investigate the degree to which health care teams were involved in quality improvement work, whereas our study concerns individuals. We intend to use psychological empowerment to understand how individual nurses and physicians are involved in managerial work more generally.

## Theoretical framework

Conger and Kanungo (1988) introduced the psychological perspective on empowerment by viewing it as a relational and motivational construct, which is the basis of our research. Maynard *et al.* (2012) claimed that, in contrast to structural empowerment, psychological empowerment focuses on individuals' perception of being in control of their work (e.g. Conger and Kanungo, 1988 ; Spreitzer, 1995 ; Thomas and Velthouse, 1990 ). Psychological empowerment means less concern with the actual transition of authority and responsibility, and instead focuses on employees' perceptions of empowerment. The basis is that individuals need to believe that they can perform their work on their own, meaning that psychological empowerment can be defined in terms of motivational processes ( Conger and Kanungo, 1988 ). The most commonly used operationalization of psychological empowerment stems from Thomas and Velthouse (1990) , who identified four cognitions of psychological empowerment that Spreitzer (1995) later refined, tested, and validated to the four dimensions of psychological empowerment at the individual level used in the present study. We connect the model's general cognitions to how nurses and physicians experience psychological empowerment with regard to managerial work:

1. Self-determination – the extent to which nurses' and physicians' sense of autonomy or control concerning the initiation or regulation of their actions can be labeled managerial work;
2. Competence – nurses' and physicians' belief in their capability to successfully perform managerial work activities;
3. Impact – nurses' and physicians' belief that they can make a difference and influence operational outcomes with managerial work and
4. Meaning – the extent to which nurses and physicians experience alignment between managerial work activities and their own beliefs.

Psychological empowerment can be described as a dynamic state or active orientation toward work, and the above-mentioned dimensions are able to capture this state/orientation ( Spreitzer, 1995 ). Spreitzer (1995) also argued that psychological empowerment is highest when all four dimensions are high, but the four dimensions are also additive. The four dimensions have been validated in several different contexts; at the individual level, Seibert *et al.* (2011) performed a meta-analysis confirming the validity. Of particular interest for this study is that the four dimensions have been validated in samples of nurses (see Boudrias *et al.* , 2004 ; Kraimer *et al.* , 1999 ).

## Method

### Design

The design of the study was motivated by the fact that while a lot of research has described *that* empowerment is important in health care, there has been less elaboration of *how* empowerment is important. Therefore, we chose to perform a qualitative interview study that enabled us to better understand the *how* question in relation to empowerment ( Silverman, 2013 ). Instead of testing hypotheses, verifying relationships between variables, etc., which has been in focus in quantitative studies, our qualitative approach enabled a more holistic and contextual understanding of empowerment in health care. Our focus was on psychological empowerment, but previous research has described structural empowerment as a necessary, yet not sufficient, antecedent to psychological empowerment ( Maynard *et al.* , 2012 ). Consequently, structural empowerment was an important condition in our selection of cases, although not our main focus.

### Case selection

Physicians have always had strong positions in health care, but nurses' empowerment is more contextual. Therefore, it was important to find cases where nurses' positions had also progressed. Consequently, we chose to perform our qualitative interview study in four primary care centers (PCCs) in Sweden, since PCCs constitute arenas in which nurses have advanced their positions considerably and most often create better structural conditions for empowerment for nurses than hospitals ( Andersson *et al.* , 2021 ; Martínez-González *et al.* , 2014 ). Our overall reason for investigating psychological empowerment for managerial work among physicians and nurses is that Glouberman and Mintzberg (2001a) described the world of physicians and nurses as being separated from the world of control/management by an abyss. Therefore, psychological empowerment among physicians and nurses for managerial work might contribute to health care integration by bridging this abyss (cf. Glouberman and Mintzberg, 2001b ).

### Data collection

The data collection was conducted through qualitative interviews with managers (5), clinical physicians (15), and clinical nurses (27) in four different PCCs. The empirical material consisted of 47 interviews, which were the total number of employees at the four PCCs who had been employed for at least one year. The interviewees are anonymized in the empirical presentation by occupational role and number. Interviews lasted between 40 and 95 min and were transcribed verbatim.

The interviews were semi-structured interviews that broadly covered the interviewees' perceptions of managerial work, covering aspects such as how the managerial work at the PCC was organized, their involvement in managerial work, their perception of managerial work, formal and informal roles, balance between managerial work and professional work, and what would make them more engaged in the PCC managerial work. The semi-structured interviews enabled follow-up questions that allowed the interviewees to expand on relevant themes and topics.

### Data analysis

The qualitative coding and analysis of data was performed in different steps, enabling both inductive and deductive analytical steps. The first step was inductive and we tried to understand how physicians and nurses were involved in managerial work. From the first inductive analysis, we saw that the most interesting results considered how non-managers related to managerial work. Therefore, in the second step we concentrated on the 42 clinical physicians and clinical nurses in the material. We then also included how they related to their clinical work, to better understand the approaches to clinical vs managerial work. We identified several themes that seemed to relate to empowerment, and especially psychological empowerment. The third step in the analytical process was to go through the empowerment literature, where we identified Thomas and Velthouse's (1990) four cognitions as especially relevant. These four cognitions were refined, tested, and validated by Spreitzer (1995) to four dimensions of psychological empowerment at the individual level. We adapted these generic cognitions into the four cognitions presented in the theoretical framework. In the fourth step, we analyzed the data material based on these four cognitions of psychological empowerment in a more deductive approach, but still aiming at qualitatively understanding than quantitatively testing. The four cognitions then constituted the structure of the results section.

## Results: nurses' and physicians' psychological empowerment for managerial work

The results section is divided into four sub-sections based on the four psychological empowerment cognitions identified previously – self-determination, competence, impact, and meaning – for managerial work among nurses and physicians.

### Self-determination

Self-determination concerns the extent to which nurses and physicians sense autonomy or control concerning the initiation or regulation of their actions that can be labeled managerial work. Regarding self-determination, it was obvious how intertwined structural empowerment and psychological empowerment are. Although our main focus was on psychological empowerment, it would be meaningless to discuss it totally separately from structural empowerment, since nurses and physicians never spoke about psychological empowerment without mentioning the structural prerequisites of empowerment.

Many of the interviewees had previous experience of a hospital context, and they often compared the PCC context with the hospital context when they spoke about it. The context seemed to influence both structural and psychological empowerment.

At a primary care center, you are more involved than at a hospital. This is my experience and I know many of my colleagues agree. Controlling processes at hospitals are always far away and out of reach. (Nurse 34)

Several quotes related to how structural empowerment led to psychological empowerment, both among nurses and physicians. Both groups had taken on actions and duties that concerned planning their work.

Together with nurses we have meetings to develop our processes. Even if the management group formally takes the decision regarding our ideas, I think we seldom meet any resistance from them. (Physician 14)

However, whereas physicians experienced psychological empowerment independently of organizational structural prerequisites (as in the quote above), nurses seemed to rely more on structural empowerment that was organizationally related as a basis for psychological empowerment:

We are two diabetes nurses here. We plan our days completely by ourselves, and not only our days; we plan everything about our patients. I enjoy working independently, we work responsibly, and our manager trusts that we manage to do that. We manage our diabetes counselling ourselves. (Nurse 24)

Therefore, managerial or other administrative positions were the main route for having a considerable influence on the actions of nurses, whereas physicians did not need to rely on the organizational structure as the basis of influence:

I don’t need to aspire for a manager position, because I think I can make a lot of decisions anyway. (Physician 10)

Physicians relied on their professional status alone as basis of structural empowerment:

Often, I have the ideas, and I check with her [the manager] whether she thinks the ideas are good, but I do what I want independent of what she thinks. (Physician 18)

Physicians could also choose to go through more organizationally related tasks to claim this autonomy/influence, but this was most often aimed at creating better conditions for their clinical work:

I think we have major possibilities to influence through the scheduling process. (Physician 14)

Another aspect of the physicians' strong professional position was that they could “allow” for managerial thinking and actions by downplaying their professional logic. For physicians, psychological empowerment for managerial actions might not necessarily be intended to perform managerial actions; it might be sufficient to not resist them.

One way to contribute [to managerial work] is by censoring myself as a doctor. Even though I would like to have something as a doctor, my understanding of available resources makes me not request things from the manager that I understand are unreasonable. (Physician 27)

To sum up the empirical results regarding self-determination, both nurses and physicians experienced psychological empowerment for managerial work, but for nurses, structural empowerment based in organizational structures was a prerequisite for psychological empowerment, whereas physicians experienced psychological empowerment independent of organizational structures. Physicians' structural empowerment was not based on organizational structures.

### Competence

Competence regards nurses' and physicians' belief in their capability to successfully perform managerial work activities. When discussing this cognition of psychological empowerment, it became obvious that clinical competence was perceived as more important than competence related to managerial work, especially among physicians, but also among nurses. However, some nurses made it very explicit how their competence regarding managerial work mattered:

I know how to structure things, and that is what it [managerial work] is about. But also to have a sense of what is coming, then you can structure with that in mind, and be ahead in planning. (Nurse 1)

It was more common for nurses to see competence in managerial work as implicit compared to their clinical competence, but they did not doubt their competence regarding managerial work.

I don’t really think of it as managerial work, but of course I plan my own activities, I control the flow of patients, and I take responsibility regarding my own time, and if you would label that managerial work, then I am good at that. (Nurse 4)

Comments from nurses seemed to question whether managerial work really required “real” competence in comparison with professional work and competence:

What would I need to become more involved in managerial work? Maybe competence [ironic laughter]. (Nurse 4)

It was common for physicians to claim that they did not have the competence, but it was not clear whether it was an excuse for choosing professional/medical tasks or if it was based in a genuine belief that they lacked competence for managerial work:

No, I am terrible at all forms of administration. I don’t think I have to know anything about the organization, I want to be a physician. (Physician 43)

In that sense, their judgment that they were not competent in managerial work was not made in isolation; it meant that they felt they were less competent at managerial work than clinical work and they thought they could contribute more to the organizations by being a physician, who was occupied with clinical work:

I take care of my available time, and I know I’m not naturally good at it [management]. (Physician 9)

Another argument was that it is hard to uphold the high level of medical competence that is required as a physician, and any managerial tasks would prevent upholding the clinical competence:

It is difficult to be both a good physician and a good manager, and my choice is clear. (Physician 29)

To sum up, nurses experienced that they were competent at managerial work. Physicians tended to describe their competence in managerial work in relation to their competence in clinical work, and felt they had less competence in managerial work. Both professions viewed their clinical competence as more important than their managerial competence.

### Impact

Impact concerns the belief of nurses and physicians that they can make a difference and influence operational outcomes with managerial work. Both nurses and physicians were mainly focused on the impact of their clinical work, but some also realized that their managerial work could influence outcomes as well, although concerning conditions rather than operational outcomes; for example, to build structures that enabled doing a better job as a clinician in the long run:

It is interesting to participate in building organizational structures that promote having right priorities from our [physician] perspective. (Physician 14)

There also seemed to be no doubt that employees did not need to become managers to influence outcomes based on managerial work.

Historically, the best improvements regarding our working processes have been initiated from the floor, not from managers. Yet, these ideas have been realized and we work accordingly, still after several years. (Physician 14)

There were also daily short-term tasks that influenced conditions of their individual professional work.

Since I am the one running my own schedule, I can influence my own working situation and promote better opportunities to do a good job. (Nurse 47)

Even if managerial work was primarily related to the conditions of the own work tasks, there were also insights that managerial tasks might influence the organization and the conditions for all employees in the longer run:

To do a good job is one thing, but that the primary care center receives compensation for this good job is another thing. To register correctly has an immense impact on our conditions to continue to do a good job. (Nurse 13)

Furthermore, some tasks had both managerial and clinical aspects, which meant integrating/accepting the managerial aspect into their clinical work influenced outcomes.

It is not always that managerial tasks are separate. For me, it is more about the fact that I am influenced by managerial thinking when performing my clinical tasks, such as thinking about costs when ordering medicine or other aid. (Nurse 13)

Otherwise, the physicians in particular often perceived an inherent conflict between managerial and clinical tasks, but did still perceive the impact of managerial tasks:

It is a daily conflict between doing things that makes a difference – clinical work – and registering what I do – managerial work. I understand that upper management is interested in what I do, and therefore I need to register it correctly, but how thorough should I be? That is the conflict. Sometimes I can accept doing stupid, administrative things, because I understand that it has a major impact on our resources. (Physician 27)

Similarly, as in regard to self-determination, physicians experienced an impact based on their professional role, independent of the task:

As a doctor at a primary care center, you have impact automatically, also when it does not concern medicine. I am not charismatic as a person, but I still have an impact only by saying something, because I am doctor. (Physician 8)

Physicians might even perceive that they would have less impact on managerial tasks if they became managers:

I might have more impact without being a manager. When the manager calls, there are always problems to be dealt with; the manager is ‘problem-marked’, whereas I am not, so I think colleagues approach me more with development issues, since I am *not* a manager. (Physician 21)

In sum, both physicians and nurses felt that they could have an impact on outcomes based on managerial work, but that managerial work was more long-term and influenced future conditions and resources more than short-term operational outcomes. Physicians experienced impact because they were physicians, feeling that they did not need to be involved in managerial work to have impact.

### Meaningfulness

Meaningfulness regards the extent to which nurses and physicians experience alignment between managerial work activities and their own beliefs. Even if the relationship (and often the conflict) between managerial work and clinical work was an aspect of all three previous psychological empowerment cognitions, it was even more clear concerning meaningfulness. For most nurses and physicians, meaningfulness in relation to clinical work was the major reason why they enjoyed their jobs. Also, the more specialized the clinical task was, the more rewarding it was.

It is my specialty reception that is most motivating, I think that is the case for most of us nurses. (Nurse 20)

To do a good job, as a doctor when meeting the patient, is all that matters. (Physician 26)

Interestingly, even nurses who had taken on manager positions experienced their clinical work as more meaningful.

The most important tasks are still clinical, despite the fact that I am a manager. I know my manager sees it differently, but I think that is most important. (Nurse 1)

It was not the managerial tasks in themselves that lacked meaningfulness; they were just perceived as less meaningful than clinical work.

Being a doctor stands in contrast with doing managerial stuff. Even though I can think it is okay to work administratively as such, it means meeting fewer patients, and that is not okay. (Physician 32)

When managerial work was considered meaningful, it often had a connection to clinical work; such as when the two were integrated:

As long as managerial work is integrated with the medical work and not something separate, it is important. (Nurse 13)

Or when managerial work created better conditions for their clinical work:

I enjoy working more independently, planning my own work. (Nurse 40)

In sum, both physicians and nurses experienced managerial work as meaningful *per se* , but it could never compete with the meaningfulness they experienced for their clinical work. Therefore, managerial work was perceived as most meaningful when it was integrated with clinical work.

Table 1 provides a comparison between physicians and nurses regarding the four cognitions of psychological empowerment.

## Discussion

This paper addresses whether clinical physicians' and clinical nurses' psychological empowerment to engage in management as part of their daily work could make managerial work and clinical work in health care organizations better integrated. Although the results of the present study are somewhat promising, it is also clear that clinicians' psychological empowerment for managerial work is far from an easy way to increased integration in health care organizations.

Previous research has described structural empowerment as a necessary, but not sufficient, antecedent to psychological empowerment ( Maynard *et al.* , 2012 ), which has also been confirmed in health care ( Lankshear *et al.* , 2013 ). In our study, it was clear that structural empowerment influenced psychological empowerment, especially regarding *self-determination* . However, there was a major difference between physicians and nurses in this respect. Whereas nurses' psychological empowerment was more conditioned, or even dependent, on structural empowerment based in organizational structures, physicians experienced psychological empowerment independently. If Maynard *et al.* (2012) are correct, physicians' structural empowerment is not based in organizational structures, but in other structures. This reflects the situation in health care with physicians as the dominant profession ( Scott, 2008 ), who obviously experience psychological empowerment not only based on their medical expertise, but as a power position that extends far beyond medical expertise to involve an institutional power. Another aspect that underscores this is that neither physicians nor nurses really perceived managerial work as being dependent on a specific *competence* , unlike their clinical work, which they described as being very much based on clinical competence. Physicians in our study seldom describe their managerial competence in isolation, but rather in relation to their clinical competence, which means that they claim more competent in clinical work than managerial work. The above-mentioned experiences of self-determination and competence are two empirical explanations of what several researchers (e.g. Bobbio *et al.* , 2012 ; Bonias *et al.* , 2010 ; Leggat *et al.* , 2010 ; Leggat *et al.* , 2011 ; McAlearney *et al.* , 2011 ) claim is needed: understanding psychological empowerment as an effect of organizational initiatives for empowerment. The present study shows that organizational structures are important for nurses when it comes to create structural empowerment that can lead to psychological empowerment, whereas physicians' strong institutional positions create structural empowerment independently of organizational structures.

Regarding the other two cognitions for psychological empowerment, *impact* and *meaningfulness* ( Spreitzer, 1995 ; Thomas and Velthouse, 1990 ), psychological empowerment for managerial work and psychological empowerment for clinical work are intertwined for physicians and nurses. Consequently, our study highlights that it is meaningless to study psychological empowerment with regard to managerial work in isolation (without relating it to psychological empowerment with regard to clinical work), because it says very little about how it influences action. Especially in terms of *meaningfulness,* physicians and nurses both perceive managerial work as meaningful *per se* , but the fact that participants from both professions perceive clinical work as *more* meaningful creates a competitive situation between the two, where experienced psychological empowerment for managerial work matters only if it does not compete with clinical work. Consequently, even if it is not conditioned that managerial work and professional work must be integrated in order for psychological empowerment for managerial work to matter, at least they should not compete. These are examples of the fine-grained aspects of psychological empowerment that constitute important contributions to existing research, which has proved *that* psychological empowerment is important for increased patient care quality and organizational effectiveness in health care (e.g. Bonias *et al.* , 2010 ; Knol and van Linge, 2009 ; Laschinger *et al.* , 2002 ), but where we now could add more knowledge about *how* it is important. Furthermore, in terms of *impact* , both physicians and nurses perceive that they can have impact on outcomes based on managerial work, but nurses in particular also seem to use the impact of managerial work to increase their impact in clinical work. Consequently, there are elements of co-optation ( Andersson and Liff, 2018 ) of managerial structures, which further emphasizes how intertwined psychological empowerment for managerial work and clinical work are. Furthermore, our study empirically confirms what conceptual papers (e.g. Andersson, 2022 ) have claimed: that managerial work and clinical work have different time frames, where clinical work is more focused on the here-and-now, while managerial work has longer time frames. Both physicians and nurses in our study claim that when they perceive outcomes of engaging in managerial work, these outcomes are related more to conditions for future work than to operational outcomes.

There is an important difference between physicians and nurses in the study. For nurses generally, engaging in managerial work is a route to structural empowerment, which leads to psychological empowerment. Organizational structures are important for nurses' structural empowerment. However, physicians do not seem to need organizational structures for structural empowerment. Their institutional position ( Scott, 2008 ) grants them structural empowerment independently of organizational structures. It is questionable whether empowerment is the route to physicians' engagement in managerial work, but engaging in managerial work has more potential for nurses.

Lastly, our study contributes with empirical findings to research that has illustrated difficulty integrating managerial and professional logics in health care (e.g. Andersson, 2015 ; Llewellyn, 2001 ; McGivern *et al.* , 2015 ). For health care clinicians to be more involved in managerial work, managerial work and clinical work do not need to be integrated *per se* , which hybrid manager research has already proven is difficult in practice ( McGivern *et al.* , 2015 ). It seems important to avoid a competitive situation between managerial and clinical work for physicians and nurses to relate to managerial tasks, but still remain knowledgeable and autonomous in their profession (cf. Noordegraaf, 2020 ). Under such conditions, psychological empowerment might be the route towards better integration of professional and managerial logics in health care organizations.

## Conclusion

This research describes and analysis how clinicians' psychological empowerment can support increased integration between management and professional thinking in health care organizations, which has important implications both for academics and practitioners. While most research has focused on empowerment in general, the present study has analyzed empowerment among physicians and nurses for specific tasks – managerial work – in relation to other tasks, namely clinical work.

The most important contribution to research is that nurses are more dependent on structural empowerment based on organizational structures to experience psychological empowerment, whereas physicians experience empowerment independently. Furthermore, the qualitative method of the study has revealed that it is not the self-determination, competence, impact, and meaningfulness in regard to managerial work *per se* that is lacking; rather, it is in competition with clinical work that managerial work is perceived as less important and meaningful. Complementing quantitative studies on empowerment with qualitative studies may create a broader understanding of the concept.

Future research could continue with qualitative studies in order to better understand how psychological empowerment is integrated with context. Otherwise, we cannot fully understand the actual consequences of psychological empowerment. Although structural empowerment is a prerequisite for psychological empowerment, it is not sufficient.

For managers in health care organizations, it is important that psychological empowerment among their clinicians can be nurtured through careful balancing between managerial and clinical work, but the previously mentioned preference for clinical work might delimit psychological empowerment with regard to managerial work if it competes with clinical values.

Practical implications of this study are that, particularly for nurses, structural empowerment based in organizational structures is an important prerequisite for psychological empowerment. For an effective health care system, structural changes in terms of positions, roles, and responsibilities can be an important route for nurses' psychological empowerment. For physicians, it seems more important the health care system is designed so there is as little competition as possible with their clinical work to enable their involvement in managerial work. The described patterns of empowerment in this study can serve as a basis for internal improvement of health care organizations that can support better health care systems.

### Limitations

Even if qualitative studies make it possible to understand contextual matters that are more difficult to capture through quantitative studies, the context dependence also creates limitations. In this case, the primary care context seems important. Most research that illustrates health care segmentation has been conducted in hospital contexts (e.g. Andersson and Gadolin, 2020 ; Andersson and Liff, 2018 ; Glouberman and Mintzberg, 2001a , b ), but PCCs tend be relatively small workplaces, with less power distance between professions, and thereby have better conditions for integration than a hospital context. Consequently, we suggest that further research investigate the same matter in hospital contexts, where segmentation might be an even bigger challenge.

## Figures and Tables

**Table 1:** Comparison between physicians and nurses

Cognitions	Physicians	Nurses
Self-determination	Strong independently of structural empowerment based in organizational structures	Conditioned or dependent on structural empowerment based in organizational structures
Competence	Relative judgment of managerial/clinical work means focusing on clinical work	Feel confident that they have the competence for managerial work
Impact	Do not need impact from managerial work, since they have impact as physicians independently	Understand that managerial work may impact the conditions for their clinical work positively
Meaningfulness	Managerial work is perceived as meaningful, but less meaningful than clinical work	Managerial work is perceived as meaningful, but less meaningful than clinical work

## Supplementary file on ethical review

The Research Ethics Committee in Gothenburg, Sweden decided that this project was not a matter for the Ethical Review Act (registration number 1126-17).
